# Fixation durations in scene viewing: Modeling the effects of local image features, oculomotor parameters, and task

**DOI:** 10.3758/s13423-016-1124-4

**Published:** 2016-08-01

**Authors:** Antje Nuthmann

**Affiliations:** Psychology Department, School of Philosophy, Psychology and Language Sciences, University of Edinburgh, 7 George Square, Edinburgh, EH8 9JZ UK

**Keywords:** Naturalistic scenes, Search, Image features, Fixation durations, Linear mixed models

## Abstract

Scene perception requires the orchestration of image- and task-related processes with oculomotor constraints. The present study was designed to investigate how these factors influence how long the eyes remain fixated on a given location. Linear mixed models (LMMs) were used to test whether local image statistics (including luminance, luminance contrast, edge density, visual clutter, and the number of homogeneous segments), calculated for 1° circular regions around fixation locations, modulate fixation durations, and how these effects depend on task-related control. Fixation durations and locations were recorded from 72 participants, each viewing 135 scenes under three different viewing instructions (memorization, preference judgment, and search). Along with the image-related predictors, the LMMs simultaneously considered a number of oculomotor and spatiotemporal covariates, including the amplitudes of the previous and next saccades, and viewing time. As a key finding, the local image features around the current fixation predicted this fixation’s duration. For instance, greater luminance was associated with shorter fixation durations. Such immediacy effects were found for all three viewing tasks. Moreover, in the memorization and preference tasks, some evidence for successor effects emerged, such that some image characteristics of the upcoming location influenced how long the eyes stayed at the current location. In contrast, in the search task, scene processing was not distributed across fixation durations within the visual span. The LMM-based framework of analysis, applied to the control of fixation durations in scenes, suggests important constraints for models of scene perception and search, and for visual attention in general.

## Introduction

Human vision during natural scene perception is an active process whereby observers selectively seek out information in the visual environment relevant to perceptual, cognitive, or behavioral goals (Findlay & Gilchrist, 2003). High-quality visual information is acquired only from the foveal region of the visual field (central ~2°). Therefore, we move our eyes about three times each second via rapid eye movements (*saccades*) to reorient the fovea around the scene. Between saccades, gaze position is relatively stable, and during these periods of *fixation*, visual information is acquired (for reviews, see Henderson, 2003; Rayner, 2009). During natural scene perception, the visuo-oculomotor system is required to make spatial decisions regarding the target location for the next saccade (i.e., the “where” decision), as well as temporal decisions regarding the time at which to terminate the current fixation (i.e., the “when” decision). The present article is concerned with the factors that influence the “when” decisions about fixation durations. Specifically, I introduce a linear mixed modeling (LMM) approach, which simultaneously considers various low-level, mid-level, and higher-level local image features, along with a number of oculomotor and spatiotemporal covariates that may affect fixation durations in real-world scene perception and search. As a second issue, I investigate how these influences depend on task-related control.

A majority of the research on eye movements during scene perception and search has focused on the “where” decision. The dominant theoretical and computational framework in the literature has been *image salience*, in which low-level image properties play a crucial role in guiding attention and the eyes (Borji & Itti, 2013; Tatler, Hayhoe, Land, & Ballard, 2011, for reviews). These models incorporate the concept of a bottom-up salience map (in differing implementations), with or without top-down control (e.g., Itti & Koch, 2000; Navalpakkam & Itti, 2005; Torralba, Oliva, Castelhano, & Henderson, 2006; Zelinsky, 2008). The scope of these models is to predict fixation locations (where), but not fixation durations (when). With regard to the “when” decision, the CRISP model is the first theoretical approach and computational model that was developed to account for variations in fixation durations during scene viewing (Nuthmann, Smith, Engbert, & Henderson, 2010). A key assumption of the CRISP model is that moment-to-moment difficulties in visual and cognitive processing can immediately inhibit (i.e., delay) saccade initiation, leading to longer fixation durations.

Empirical studies on the “where” decision have addressed the question of which image characteristics predict where people fixate when viewing natural images (e.g., Baddeley & Tatler, 2006; Mannan, Ruddock, & Wooding, 1996; Reinagel & Zador, 1999; Tatler, Baddeley, & Gilchrist, 2005). Nuthmann and Einhäuser (2015) combined a scene-patch analysis with generalized linear mixed models (GLMMs). Using this method, the authors estimated the unique contributions of various image features to fixation selection: luminance and luminance contrast (low-level features), edge density (a mid-level feature), and visual clutter and image segmentation, to approximate local object density in the scene (higher-level features). The GLMM results revealed that edge density, clutter, and the number of homogeneous segments in a patch can independently predict whether or not image patches are fixated. Importantly, neither luminance nor contrast had an independent effect above and beyond what could be accounted for by the other image features (Nuthmann & Einhäuser, 2015).

### “When” decision about fixation duration

More recently, interest has been growing in the oculomotor decision of when to move the eyes during scene viewing (e.g., Glaholt & Reingold, 2012; Henderson & Pierce, 2008; Nuthmann et al., 2010; Pannasch, Schulz, & Velichkovsky, 2011). The underlying idea is that fixation durations in visual-cognitive tasks vary with processing difficulty (Rayner, 1998). In line with this general assumption, fixation durations during scene viewing have been shown to globally adjust to overall processing difficulty. Importantly for the present study, image-wide degradations of low-level features have been shown to prolong fixations. In one set of studies, image features were manipulated throughout the entire viewing period of the scene, and fixation durations were prolonged when the overall luminance of the scene was reduced (see below) or when color was removed (Ho-Phuoc, Guyader, Landragin, & Guerin-Dugue, 2012; Nuthmann & Malcolm, 2016). Fixation durations also increased when high-spatial-frequency information was removed through low-pass filtering (Mannan, Ruddock, & Wooding, 1995), or when higher-order scene statistics, including objects, were removed (Kaspar & König, 2011; Walshe & Nuthmann, 2015).

In addition, studies using gaze-contingent display-change paradigms have tested the direct-control hypothesis, which states that the processing of the scene stimulus currently in view produces an immediate fixation-by-fixation adjustment of the timing of the saccade that terminates the fixation (Rayner & Reingold, 2015, for a review focusing on reading). The scene-onset delay (SOD) paradigm (Henderson & Pierce, 2008; Henderson & Smith, 2009; Luke, Nuthmann, & Henderson, 2013; Shioiri, 1993) offers the most straightforward approach for demonstrating that the information extracted during a fixation impacts the timing of the saccade terminating that fixation. At the beginning of a critical fixation, a visual mask is presented, which delays the onset of the scene. The duration of the delay is varied. The scene is then presented normally until the observer looks at another scene region. The underlying rationale is that stimulus processing can only begin after the visual features of the stimulus have become available. Indeed, SOD studies have consistently revealed populations of fixations that increased in duration as the delay increased, suggesting that the durations were controlled directly and in real time by the current scene image. Simulations with the CRISP model substantiated that for these fixations, the initiation of a new saccade program is delayed due to the stimulus’s unavailability at the beginning of a fixation, resulting in an increase in fixation durations (Nuthmann & Henderson, 2012; Nuthmann et al., 2010). Further evidence in support of direct control has been provided by the fixation-contingent scene quality paradigm, in which the quality of the scene is manipulated during the entire duration of selected critical fixations (Glaholt, Rayner, & Reingold, 2013; Henderson, Nuthmann, & Luke, 2013; Henderson, Olejarczyk, Luke, & Schmidt, 2014; Walshe & Nuthmann, 2014a). In these studies, image-wide feature modifications have been used as a means to degrade or enhance the scene stimulus. The durations of the critical fixations were immediately affected by reductions in luminance (see below) or by filtering high or low spatial frequencies (Glaholt et al., 2013; Henderson et al., 2014). Collectively, these findings lend support to the notion that fixation durations are, at least partially, under the direct moment-to-moment control of the current visual stimulus.

All these experiments have in common that the *entire* scene was manipulated, to vary *global* scene processing difficulty. The present work extends this line of research by investigating *local* effects of image features on fixation durations under different task instructions. Specifically, the present study combines a corpus analysis approach with an experimental manipulation. The aim of the study was to collect a large corpus of eye movements from a large number of participants (*N* = 72) viewing a large number of scenes (*N* = 135). In addition, the observers’ viewing task (scene memorization, preference judgment, or scene search) was manipulated as part of the study design. This was done to investigate how the control of fixation durations depends on cognitive top-down influences in addition to a putative role of bottom-up image features.

With regard to local image features, the corpus analyses considered the sets of low-level, mid-level, and higher-level visual image features used in a related study on fixation selection in scenes (Nuthmann & Einhäuser, 2015). For a particular image and/or fixation location, different features tend to be correlated (Baddeley & Tatler, 2006). Although feature dependencies can be a consequence of the hierarchical definition of features, they oftentimes arise from the structural properties of natural scenes (Nuthmann & Einhäuser, 2015). To deal with feature dependencies, I used an LMM-based statistical control approach to assess each feature’s unique contribution to fixation duration. The main focus was on testing whether local image statistics exert *immediacy effects* on fixation durations in scene viewing. For example, does the luminance in a limited spatial region around the current fixation modulate this fixation’s duration? In addition, the analyses focused on whether scene processing is distributed across fixation durations within the visual span, an idea first proposed in research on eye movements in reading (e.g., Engbert, Nuthmann, Richter, & Kliegl, 2005; Kliegl, Nuthmann, & Engbert, 2006; Schad, Nuthmann, & Engbert, 2010). This approach implied testing whether the duration of the current fixation also reflected the processing demands of the previous and next fixation locations. Along with the image-related predictors, the LMMs simultaneously considered a number of oculomotor and spatiotemporal covariates. Separate models were built for the three different viewing tasks. In the remainder of this introduction, I will introduce the variables that are part of the analysis framework in more detail. Where relevant, the results from reading studies will be presented along with findings from scene-viewing studies.

### Viewing task

Task effects have provided compelling demonstrations of the cognitive top-down influences on eye movements in scene viewing (Yarbus, 1967). On the basis of a subset of the present data (36 participants, two tasks), we previously reported longer fixation durations in a memorization task that probed scene memory, as compared with an object-in-scene search task (Nuthmann et al., 2010). This global effect of viewing task on fixation durations was modeled with the CRISP model (Nuthmann et al., 2010), with task-specific influences being realized by different parameter settings. Castelhano et al. (2009) compared a memorization task probing memory for objects in scenes with a search task in which participants were asked to locate a specified object in the scene. There were no differences in individual fixation durations between the two experimenter-directed task manipulations. However, longer gaze durations were observed on objects in the scenes during memorization than during search. In a study by Mills et al. (2011), participants completed one of four tasks (memory, pleasantness, search, or free view) under general viewing instructions that were participant-directed (i.e., the task instructions established general goals of viewing and left the participants free to translate them). The task set biased the timing of fixations, such that fixation durations were generally longer for free view and memory than for search and pleasantness judgment.

### Image features

For every image location that observers sampled with their eye fixations, five local image-based indexes of processing difficulty were obtained. First, three common measures of local image statistics that characterize different properties of image luminance were examined: luminance, luminance contrast, and edge density. In addition, the effects of the processing load induced by the two more complex, higher-level image-based measures were evaluated. Specifically, the feature congestion measure of visual clutter (Rosenholtz, Li, & Nakano, 2007) was included as a surrogate measure for objects, and synergistic image segmentation (Christoudias, Georgescu, & Meer, 2002) as an approximation of local object density in the scene. A few studies have considered the association between fixation duration and individual measures, using experimental or correlational methods.

#### Luminance

It has been shown that reducing the luminance of the entire scene leads to longer fixation durations (Henderson et al., 2013; Loftus, 1985; Loftus, Kaufman, Nishimoto, & Ruthruff, 1992; Walshe & Nuthmann, 2014a). For example, in the Henderson et al. study, participants freely viewed scenes at three levels of luminance (100 %, 80 %, and 60 %) in preparation for a later memory test. In a first experiment, each scene was presented at one of the luminance levels for the entire trial, and fixation durations linearly increased as luminance decreased. Thus, fixation durations were *globally* slowed when scene processing became more difficult. In two additional experiments, scenes were reduced in luminance during saccades ending in critical fixations. The duration of these critical fixations was *immediately* affected by the reduction in scene luminance, with increasing durations for decreasing luminance. Walshe and Nuthmann (2014a) replicated and extended these results, and then modeled the key findings with a variant of the CRISP model (Walshe & Nuthmann, 2014b).

#### Luminance contrast

Einhäuser and König (2003) had five participants view outdoor scenes without any visible manmade objects; no task-specific instructions were given. The duration of fixations was correlated with neither contrast nor experimental contrast modifications.

#### Clutter

Clutter is an image-based feature of visual complexity, which has been studied mostly in the context of a search task. Rosenholtz et al. (2007) operationalized clutter using three image-based measures: feature congestion, sub-band entropy, and edge density (see below for details). With regard to fixation durations, it may be expected that a more cluttered scene would lead to longer average fixation durations. Henderson et al. (2009) tested this hypothesis by reanalyzing data from a difficult scene search task. Fixation durations were influenced by global scene clutter within the first second of search (significant correlations with all three measures of scene clutter), but not by the local clutter surrounding the current fixation location (square regions 1° or 3.3° in size), a counterintuitive finding.

### Distributed-processing assumption: Lag and successor effects

**Fig. 1:**
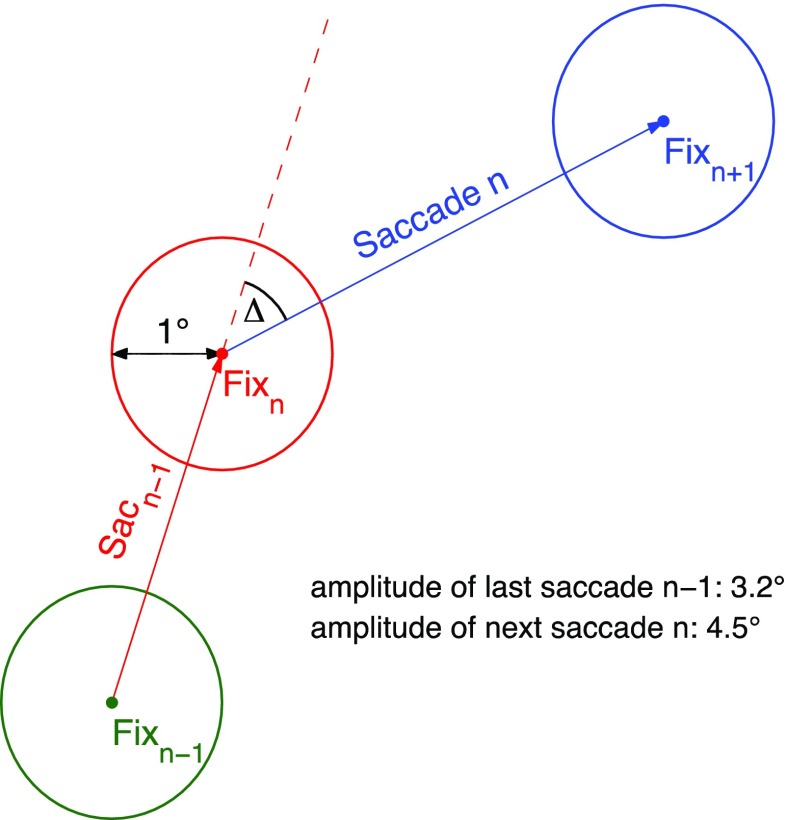
Schematic depiction of the analyzed fixation triplets. The dependent variable in all analyses is the duration of the middle fixation *n* (Fix_*n*_). This current fixation *n* is preceded by saccade *n –* 1 (Sac_*n*–1_), which moved the eyes from the previous fixation *n –* 1 to the current fixation location *n*. The next saccade *n* moves the eyes from fixation *n* to the next fixation location, *n* + 1. Δ denotes the angular difference between the previous saccade *n* – 1 and the next saccade *n*. The circles around the fixation locations depict the patches that were used to test the influences of visual image features

Evidence that observers are able to process parafoveal information during scene viewing has been provided by visual-span studies. The *visual span* (also referred to as the *perceptual span*) is defined as the area of the visual field from which useful information can be acquired during a given eye fixation (see Rayner, 2009, 2014, for reviews). The size of the visual span can be measured using the gaze-contingent moving-window paradigm (McConkie & Rayner, 1975). The general logic is to reduce the size of the window to find the smallest window that still supports normal scene-viewing behaviors. The size of the visual span in scene viewing is large, encompassing up to half of the total scene (scene search: Nuthmann, 2013; scene memorization: Saida & Ikeda, 1979). For object-in-scene search,^1^ the visual span corresponded to 8° in each direction from fixation (Nuthmann, 2013). When the radius of the high-resolution moving window was smaller than 5°–6° (fixation-duration-based visual span size), the fixation durations systematically increased. Conversely, we can infer from these findings that visual information within both foveal (~1° eccentricity) and parafoveal (~5° eccentricity) vision can influence fixation durations. This opens up the possibility that scene processing may be distributed across fixation durations within the visual span (*distributed-processing assumption*). Thus, the starting point for my investigation was that scene-level features can be processed across the visual field. I then tested the distributed-processing assumption in two steps. First, I tested whether there are *immediacy effects* of local image statistics on fixation durations in scene viewing. For example, does the luminance or clutter around fixation modulate fixation durations? Second, I tested whether the duration of the current fixation also reflects the processing demands of the previous fixation location (*lag effect*, *spillover effect*) or the next (*successor effect*, *parafoveal-on-foveal effect*).^2^ Therefore, the analyses considered triplets of fixations—that is, sequences of three successive fixations (Fig. 1). The current fixation is referred to as fixation *n*, the preceding fixation as *n* – 1, and the next fixation as *n* + 1. The only dependent variable was the duration of fixation *n*. To test the local influence of visual image features, circular image patches with a radius of 1°, approximating foveal vision, were centered on each fixation point.

*Lag effects* refer to the influence of local image-based indexes of fixation *n* – 1 or the position of fixation *n* – 1 on the duration of fixation *n*. Corpus analyses of reading data have identified lag effects that are (a) due to incomplete processing of the previous word *n* – 1 and (b) due to the limits of visual acuity (Kliegl et al., 2006). The present analyses tested whether lag effects originating from these two sources also exist in scene viewing. First, if the processing of the scene region sampled with fixation *n* – 1 is not completed before the eyes move on to the next scene region, effects of image statistics at fixation *n* – 1 might spill over to the duration of the subsequent fixation *n*. Second, the distance between the locations of fixations *n* and *n* – 1—that is, the amplitude of the incoming saccade—might also influence the subsequent fixation duration. In reading, the finding that fixation durations increase with the amplitude of the incoming saccade is well-established (e.g., Kliegl et al., 2006; Schad et al., 2010; Vitu, McConkie, Kerr, & O’Regan, 2001). Likewise, in scene viewing we may observe long fixations after long saccades because the previous fixation *n* – 1 yielded less preview of the scene region sampled with the current fixation *n* than is true for fixations after short saccades. In free viewing, when there is no explicit task, the amplitude of the incoming (or last) saccade (Sac_*n*–1_ in Fig. 1) has not predicted the duration of the following fixation (Tatler & Vincent, 2008).^3^ To foreshadow the results, I found systematic effects of saccade amplitude on subsequent fixation durations across viewing tasks in the present data.

*Successor effects* refer to the possibility that processing of scene regions in parafoveal vision can influence foveal fixation durations during scene viewing. Parafoveal information is used to provide information as to where the eyes should move next (Nuthmann, 2013; Pajak & Nuthmann, 2013). Specifically, this information is used for selecting the next saccade target and determining the amplitude of the next saccade. However, it is currently unclear whether and to what extent such parafoveal processing modulates the duration of fixation *n*. Do successor effects generalize from reading (Kliegl et al., 2006; Schotter, Angele, & Rayner, 2012, for a review) to scene viewing? If so, is the parafoveal processing of upcoming fixation locations restricted to low-level properties related to image luminance, or does it also extend to higher-level image features that approximate the presence of objects in a scene? Finally, do successor effects depend on task-related control?

### Oculomotor and spatiotemporal parameters

Along with the image-related predictors, the LMMs simultaneously assessed a number of oculomotor and spatiotemporal covariates, including the amplitude of the next saccade, the change in saccade direction, and viewing time.

#### Amplitude of the next saccade

The LMMs included the amplitude of the outgoing (or next) saccade. Tatler and Vincent (2008) found no systematic relationship between the current fixation duration and the amplitude of the outgoing saccade (Saccade *n* in Fig. 1) during free viewing of natural scenes. Reading studies have reported mixed results. In a number of studies, fixation durations were found to increase with the length of the outgoing saccade (e.g., Kliegl et al., 2006; Kuperman, Dambacher, Nuthmann, & Kliegl, 2010; Schad et al., 2010). However, corpus analyses by Angele et al. (2015, 2016) reported significant negative effects, with shorter single fixations and gaze durations when the next saccade was large.

#### Change in saccade direction

The change in saccade direction can be described as the angular difference between the last saccade *n* – 1 and the next saccade *n* (Δ in Fig. 1). An angle Δ = 0° is indicative of a saccade *n* that continues the trajectory of saccade *n* – 1, whereas Δ = 180° denotes a complete reversal of direction. A number of studies have observed an approximately linear increase in fixation duration and/or saccade latency as a function of the angular difference between the last and next saccades (Klein & MacInnes, 1999; MacInnes & Klein, 2003; Smith & Henderson, 2009, 2011; Tatler & Vincent, 2008; Wilming, Harst, Schmidt, & König, 2013). Fixation durations are shortest when saccade *n* continues the trajectory of saccade *n* – 1, whereas complete reversals in saccade direction are associated with the longest fixations. In the literature (see Klein & Hilchey, 2011, for a review), the effect has been associated with the temporal component of either (or both) of two biases: a bias away from previous fixations (i.e., *oculomotor inhibition of return*, O-IOR) or a bias for the eyes to continue moving in the same direction (i.e., *saccadic momentum*).

#### Viewing time

The finding is well-established that fixation durations change over time. Several studies have reported that fixation durations increased during initial viewing periods and stabilized during later viewing (e.g., Antes, 1974; Mills et al., 2011; Pannasch, Helmert, Roth, Herbold, & Walter, 2008; Unema, Pannasch, Joos, & Velichkovsky, 2005; but see De Graef, Christiaens, & D’Ydewalle, 1990). The study by Mills et al. (2011) investigated how task set influences the rate of change in fixation durations over the course of viewing. As was described above, fixation durations were generally greater for free view and memory than for search and pleasantness rating. The effect was present primarily during early viewing only (i.e., at 1 and 2 s), with the only difference during later viewing (i.e., at 5 s) being between the free-view and the search conditions (Mills et al., 2011). In contrast, in the study by Castelhano et al. (2009), no effect of task (memorization vs. search) was observed across the viewing period or during early viewing (the first five fixations).

#### Distance from scene center

Many studies have reported that observers fixate more often toward the center of the image than at the edges (e.g., Mannan et al., 1996; Tatler et al., 2005). This image-independent viewing bias (Tatler, 2007) is referred to as the *central bias of fixation*. In previous work, this bias has been quantified as a linear decrease in fixation probability as the distance from scene center increases (Nuthmann & Einhäuser, 2015). When the influence of image features was controlled for, the central bias was still a strong predictor of where observers fixated in a scene. To explore whether the central bias generalizes to fixation durations, the current fixation’s spatial distance from image center was considered as an additional input variable for analysis.

### The present study

The present research aims at advancing our knowledge about the factors that control fixation durations during scene viewing. This study is the first to present a statistical modeling framework to simultaneously test the influences of a large set of variables on fixation durations during scene perception, with a specific focus on how local image-based indexes of processing difficulty influence the fixation durations at the current, previous, and next fixation locations. An LMM approach is introduced, which allows the researcher to assess each predictor’s unique contribution to explaining variance in fixation durations for a given viewing task, and its relative importance. Specifically, the goal of the LMMs was to test simultaneously the influences of 20 variables. These are the luminance, luminance contrast, proportion of edges, visual clutter, and number of segmented units around the current, previous, and next fixation locations; the amplitudes of the incoming and outgoing saccades (in degrees of visual angle); the angular difference between the two saccades (in degrees); the current fixation’s Euclidian distance from image center (in degrees of visual angle); and the viewing time (in milliseconds).

## Method

### Participants, apparatus, and materials

Analyses were based on a large corpus of eye movements during scene viewing.^4^ Seventy-two participants (mean age = 22.6 years; 38 females, 34 males) each viewed 135 unique full-color photographs of real-world scenes from a variety of categories (indoor and outdoor). The 92 indoor scenes came from different subcategories, ranging from common rooms in one’s house (e.g., living room, kitchen) to images from shops, garages, and so forth. Scene images were presented on a 21-in. CRT monitor with a screen resolution of 800 × 600 pixels. The scenes subtended 25.78° horizontally × 19.34° vertically at a viewing distance of 90 cm. A chinrest with a head support was used to minimize head movement. During scene presentation, eye movements were recorded using an SR Research EyeLink 1000/2 K system (average accuracy: 0.25° to 0.5°, precision: 0.01° root-mean squared). The experiment was implemented with the SR Research Experiment Builder software.

### Procedure

**Fig. 2:**
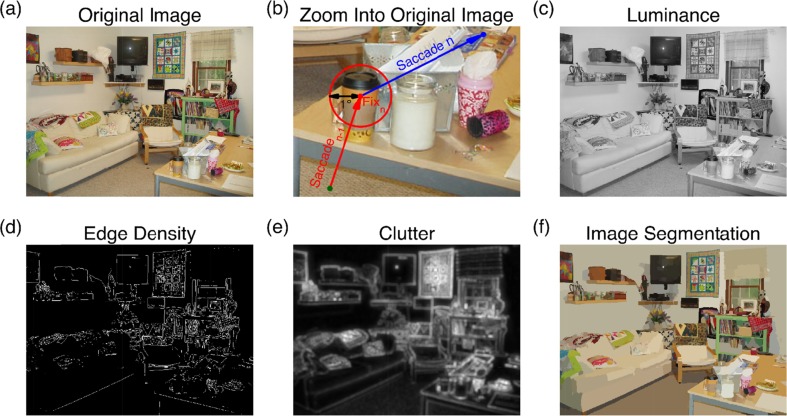
Example image and feature maps. (a) The original image. (b) Zooming in to the table on the lower right, with a fixation on the paper coffee cup. (c) Luminance map. (d) Edge density map, after filtering the image with a Sobel operator. (e) Feature congestion visual clutter map. (f) Synergistic segmentation of the scene image, resulting in 2,277 homogeneous tiles

Participants viewed each of the 135 scenes once: 45 scenes in each of the three viewing tasks (memorization, preference judgment, and search). All scenes were presented for 8 s. In the scene memorization task, observers had to encode the scene to prepare for an old–new recognition test administered at the end of the experiment. In the aesthetic preference judgment task, participants rated how much they liked each scene. The visual search task had participants look for a prespecified object in the scene (e.g., the basket in Fig. 2a).

At the beginning of each trial, a fixation point was presented at the center of the screen and acted as a fixation check. In the search task, prior to the fixation check, a text label describing the target (e.g., *basket*) was presented for 800 ms. For details on selection of the search targets and their properties, see Nuthmann and Henderson (2010). To keep the viewing time constant across tasks, the scene remained on the screen until the 8 s were over. However, the present fixation duration analyses only considered fixations made until the buttonpress terminating the search.

Both the search block and the aesthetic preference block were preceded by three practice trials. After participants had completed the three viewing tasks, the memory test was administered (see Nuthmann & Henderson, 2010, for details).

### Design

A dual Latin-square design was used in the study (Table 3). Participants were allocated to nine groups of eight participants (random factor Subject Group) to control for (a) which set of images they viewed in each task and (b) the order in which they performed the three viewing tasks. To control for item effects, the 135 scene images were assigned to three lists of 45 scenes each. The scene lists (random factor Scene List) were rotated over participants, such that a given participant was exposed to a scene list for only one of the three viewing-task conditions. The three orders in which the task blocks were completed were search–memorization–preference, preference–search–memorization, and memorization–preference–search. The design ensured that every order of tasks and combination of scenes with tasks was represented at least once across the nine participant groups (Table 3). Out of the 72 participants, 24 saw the same scene images in a given viewing task, and eight participants saw the same images in a given task and task order.

### Data analysis

Data from the right eye were analyzed. Saccades were defined with a 50°/s velocity threshold using a nine-sample saccade detection model. The raw data were converted into a fixation sequence matrix using SR Research Data Viewer. Those data were processed further and analyzed using MATLAB 2009b (The MathWorks, Natick, MA) and the R system for statistical computing (version 3.2.0; R Development Core Team, 2015) under the GNU General Public License (Version 2, June 1991). All image processing was performed in MATLAB.

#### Gaze data analysis

A major goal of the present work was to test the influences of local image-based indexes of processing difficulty on the fixation durations at the current, previous, and next fixation locations. Therefore, the main analyses considered triplets of fixations (Fig. 1). Fixations were excluded if they were the first or last fixation in a trial. The triplet analyses therefore required a minimum of five fixations in a trial. To test the influences of visual image features, circular image patches were centered on each fixation point. Each circle had a radius of 1°, to approximate foveal vision while accommodating the inaccuracy of the eyetracker. A given fixation could potentially contribute to several triplets. For example, a sequence of five successive valid fixations would generate three triplets, with the middle fixation (#3) contributing to the first triplet as fixation *n* + 1, to the second triplet as fixation *n*, and to the third triplet as fixation *n* – 1. Fixation triplets that co-occurred with blinks were removed. For the investigation of fixation durations, it is common to exclude very short (e.g., <50 or 80 ms) and very long fixations, on the basis of the assumption that they are not determined by online cognitive processes (Inhoff & Radach, 1998). Triplets in which one or more fixations were shorter than 50 ms or longer than 1,000 ms were therefore disregarded. For the investigation of saccade properties, it is common to remove saccades with amplitudes less than 1°, to exclude corrective saccades and microsaccades (e.g., Smith & Henderson, 2009). In the present context, the inclusion of small saccades would potentially smear out the effects of distributed processing. Furthermore, the length of the next saccade from fixation *n* to *n* + 1 determined the overlap between the circular patches centered on fixations *n* and *n* + 1, and the same was true for the previous saccade and the patches centered on fixations *n* – 1 and *n*. When using circular patches with a 1° radius, a 1° saccade would lead to a 39 % overlap between neighboring patches. Overlap between patches would also aggravate the correlation between them. Only fixation triplets in which the circular patches around fixations *n* – 1, *n*, and *n* + 1 did not overlap were included in the analyses. Triplets in which the incoming or outgoing saccades (or both) were shorter than or equal to 2° were therefore removed. After exclusions, 76,685 fixation triplets (memorization: 28,442; preference judgment: 33,275; search: 14,968) remained for the analyses. Fewer data points were available for the scene search task because the analyses only considered fixations made until the buttonpress terminating the search (mean search time 3.77 s). The median saccade amplitudes were 5.1° (search), 5.2° (memorization), and 5.3° (preference).

The triplet analyses required filtering the data set in various ways, such that the analyses were based on subsets of data. Therefore, the triplet analyses were complemented by control analyses that exclusively tested immediacy effects of local image features around the current fixation—that is, no lag and successor effects. As before, the LMMs included the full set of oculomotor and spatiotemporal variables. As compared with the triplet analyses, the number of observations that entered the control LMMs was much increased (memorization: 67,472; preference judgment: 69,854; search: 33,170), thereby increasing statistical power. Moreover, the control analyses allowed for testing whether the results would generalize when fixations with short incoming or outgoing saccades were included.

#### Computation of image features

For each scene image, five different feature maps were calculated. On the basis of the various image feature maps, local image statistics were calculated by identifying patches subtending a circular area with a radius of 1° (31 pixels) around fixation locations. Patches were computed for each participant and scene on a fixation-by-fixation basis. Thus, the local image patches were analyzed for all three fixations in a triplet (Fig. 1).

##### Luminance

The luminance of each pixel was defined by converting the sRGB values of the image (assuming the IEC 61966-2-1 specification) to CIE L*a*b* space and retaining only the luminance (L*) information. For each image, luminance was then mapped linearly to the interval [0, 1]. As an illustration, Fig. 2c depicts the luminance map for the example scene in Fig. 2a. Local luminance was defined as the mean value of the luminance within a patch. Greater luminance is associated with a higher degree of subjectively perceived brightness.

##### Luminance contrast

On the basis of the luminance map (Fig. 2c), each local image patch was labeled with its local contrast value. The contrast for each patch was defined as a version of the root-mean-square contrast (Moulden, Kingdom, & Gatley, 1990): that is, the standard deviation of the luminance values of all pixels in the patch, divided by the mean luminance of the image (Einhäuser & König, 2003; Reinagel & Zador, 1999). In general, more uniform patches have less contrast.

##### Edges

Edges were defined as the boundaries between regions of distinctly different mean luminances. The locations of the edges in an image were determined by applying a Sobel operator to the luminance map, which extracted an approximation to the luminance gradient at each point in the image (Mannan et al., 1996; Mannan, Ruddock, & Wooding, 1997). Thresholds were applied using the adaptive procedure implemented in the *edge* function in the Image Processing Toolbox for MATLAB, resulting in a binary image with ones where the function found edges in the image and zeros elsewhere. The procedure thus produced a black-and-white image, with white representing the edges (see Fig. 2d). Edge density was then defined as the mean over all pixels in a patch for this binary image; that is, the proportion of edges in the patch. These proportions ranged from 0 to .374 (mean = .068, standard deviation = .043). To “stretch out” proportions that are close to 0, edge densities were submitted to a logit transformation [logit(*p*) = 0.5 * ln(*p*/(1 – *p*))] (Cohen & Cohen, 1975), after regularizing 0 to the smallest nonzero value in the data.

##### Clutter

A feature congestion map of visual clutter was computed for each scene, using the algorithms described by Rosenholtz et al. (2007) and MATLAB code provided at http://dspace.mit.edu/handle/1721.1/37593. For each such feature map, the range of feature values was normalized linearly to [0, 1]. Figure 2e depicts the feature congestion map of visual clutter for the example scene shown in Fig. 2a. The local feature values for clutter were defined as the mean over this feature map’s values within each patch.

##### Synergistic image segmentation

The goal of image segmentation is to break up the image into meaningful or perceptually similar regions. The present analyses used the synergistic segmentation (Christoudias et al., 2002), which combines the mean shift-based color image segmentation (Comaniciu & Meer, 2002) with edge confidence and gradient maps (Meer & Georgescu, 2001). The algorithms, implemented in C++, are available via the Edge Detection and Image Segmentation (EDISON) system (Christoudias et al., 2002), as is a MEX wrapper for MATLAB (www.wisdom.weizmann.ac.il/~bagon/matlab.html). Each image was subjected to the synergistic image segmentation by using the default parameters. On average, 2,947 segments per scene were obtained (see Fig. 2f for an example). For each patch, the number of homogeneous segments was determined.

#### LMMs and model-building strategy

LMMs (e.g., Baayen, Davidson, & Bates, 2008) were used to determine the impacts of various image-based, oculomotor, and spatiotemporal variables on the fixation durations in scene viewing. The main focus was on the effects of local image statistics. Therefore, and to reduce model complexity, models were built separately for each of the three viewing tasks. This approach allowed for inferences about the presence or absence of a given effect in a given viewing task. Whereas one can assess the strength of a given effect in a given task through the size of the standardized regression coefficient (Schielzeth, 2010; Schielzeth & Forstmeier, 2009, for discussion in the context of LMMs), the effect of viewing task is not explicitly modeled. Moreover, given that this was the first study of its kind, the models reported here will include main effects without interactions.

Mixed models are statistical models that incorporate both fixed and random effects (Bates, 2010). *Fixed effects* in LMM terminology correspond to regression coefficients in standard linear regression models or to main effects in an analysis of variance.^5^ *Random effects* allow for capturing variance attributed to the randomness of participant and item sampling. The participants and items tested in research on scene perception are crossed, in that the participants in a given study are tested on a series of scene items, and the same items are tested on a series of participants. Technically, random effects represent the subjects’ or items’ deviations from the fixed-effect parameters (Bates, 2010).

The many advantages of LMMs are well-documented (Cunnings, 2012; Judd, Westfall, & Kenny, 2012; Kliegl, Wei, Dambacher, Yan, & Zhou, 2011; Locker, Hoffman, & Bovaird, 2007). An important advantage is that LMMs allow one to generalize to populations of both subjects and items on the basis of a single analysis. Another advantage is that they avoid information loss due to prior averaging over items or subjects. In the present context, this means that fixation durations were modeled on the fixation level. Moreover, LMMs can handle incomplete and unbalanced data, an inherent feature of many eyetracking studies.

It is important to distinguish between input variables and predictors. *Input variables* are the variables that were measured, and *predictors* are the terms that were entered in the model (Gelman & Hill, 2007; Schielzeth, 2010). Here, all input variables were measured on a continuous scale. For the LMM analyses, all input variables were centered by subtracting the sample mean from all variable values, and scaled by dividing the variables by their sample standard deviations. As a result, each input variable had a mean of 0 and a standard deviation of 1. This standardization (*z* transformation) converts the original units to units of standard deviations. In the case of approximately normally distributed input variables, about 95 % of the values are within ±2 units. The standardization of input variables results in the estimation of standardized slopes, which are comparable in magnitude within models as well as between models (Schielzeth, 2010). Fixation durations were log-transformed to achieve a near-normal distribution of the dependent variables (see Kliegl, Masson, & Richter, 2010) and to avoid issues with heteroscedasticity.

When analyzing empirical data with LMMs, the selection of an appropriate random-effects structure is of key importance. In the LMMs reported here, the fixed-effect intercept reflects the mean fixation duration (log-transformed) in a given viewing task. The intercept has several random components. The first one varies from subject to subject, to allow for the fact that some observers have longer fixation durations on average than others. Including such by-subject random intercepts is also a way of accounting for individual differences. The second random component for the intercept varies from scene item to scene item. In the design of the study, the great variation in the composition of natural scenes was accounted for by counterbalancing scene lists across viewing-task conditions. However, individual scene items may have effects above and beyond their affiliations with certain item lists. In the context of LMMs, this was accounted for by including by-item random intercepts (and slopes; see below). For completeness, the random-effects structure of the LMMs also included random intercepts for the factors Scene List and Subject Group (see the Method section), following the Latin-square example in Baayen et al. (2008).

In principle, the variance–covariance matrix of the random effects not only includes random intercepts but also random slopes, as well as correlations between the intercepts and slopes. Random slopes account for variance between subjects and between items for the fixed effects (and interactions) in the model. For example, the by-item random slope for edge density at fixation *n* captures whether items vary in the extents to which they show effects of foveal edge density on fixation durations.

Models that include random intercepts but no slopes (i.e., random intercept models) can lead to false positives, such that the null hypothesis regarding an experimental effect is wrongly rejected (Schielzeth & Forstmeier, 2009). Simulation studies have shown that LMMs minimize the false positives when they include the maximal random-effects structure justified by the design (Barr, Levy, Scheepers, & Tily, 2013). The problem with the maximal random-effects structure is that the number of model parameters associated with the random factors grows quadratically with the number of variance components (Bates, Kliegl, Vasishth, & Baayen, 2015a). Specifically, for *n* variance components there will be a maximum of *n*(*n* + 1)/2 model parameters (not counting fixed effects).

As was outlined in the introduction, LMMs with the full fixed-effects structure considered 20 input variables. Two of the input variables entered the model with a quadratic term in addition to the linear term (i.e., amplitude of the previous saccade and viewing time). Along with the intercept, the models therefore comprised 23 fixed effects. The maximal random-effects structure would require estimating 554 parameters (by subject, a random intercept, 22 random slopes, and 253 correlation terms; by item, the number was the same as by subject; two additional random intercepts). Needless to say, this maximal random-effects structure is too complex for the information contained in the data, with the result that the LMM would not converge.

One recommendation to reduce model complexity is to set correlation parameters to zero (Barr et al., 2013; Bates, Maechler, Bolker, & Walker, 2015b). Thus, in such a zero-correlation parameter model (zcpLMM), the random slopes and intercepts are assumed to be independent. Given the relatively large number of fixed effects, the full random-effects structure of the zcpLMM is still complex, requiring 48 variance components to be estimated (23 by subject, 23 by item, plus two additional random intercepts). These models, one for each viewing task, did converge after a large number of model evaluations (26,165, 28,329, and 19,288 iterations for the memorization, preference, and search tasks). Whether random effects are warranted for a given fixed effect is an empirical question (Judd et al., 2012). The estimates for the variance components in the zcpLMMs (with a full random-effects structure) revealed that the variances for a number of random slopes were estimated as zero. The majority of them were by-subject random slopes related to the local image features around fixations *n* – 1 (memorization, two out of five; for preference, four; and for search, three), *n* + 1 (for memorization four, for preference one, and for search three), and fixation *n* (for memorization zero, for preference four, and for search three).^6^ Therefore, the complexity of the zcpLMMs was reduced by excluding all by-subject random slopes pertaining to local image features. In addition, no evidence was apparent that subjects or items varied with regard to the quadratic term for the amplitude of the previous saccade. Consequently, random slopes for the amplitude of the previous saccade were limited to the linear term. The resulting final models comprised 31 variance components.

For model parameter estimation, restricted maximum likelihood (REML) estimation was used. For model comparisons, the models were refit using the maximum likelihood criterion (Bates, 2010). For the fixed effects, the coefficient estimates (*b*), their standard errors (*SE*), and *t* values (*t* = *b*/*SE*) are reported. There is no clear definition of “degrees of freedom” for the error term in LMMs, and, therefore, precise *p* values cannot be estimated (Baayen et al., 2008). The LMMs reported in the present article were based on a large number of observations, participants, and scenes, and included a comparatively small number of fixed and random effects. In such a case, the *t* distribution is equivalent to the normal distribution for all practical purposes, so that the contribution of the degrees of freedom to the test statistic is negligible (Baayen et al., 2008, note 1). Therefore, a two-tailed criterion (|*t*| > 1.96) was used to determine significance; effects with |*t*| > 1.645 indicated marginal significance (cf. Schad et al., 2010).

The LMM analyses were computed in R, using the lmer program of the lme4 package (version 1.1-8; Bates et al., 2015b). Figures depicting predicted partial effects on fixation duration were created using the ggplot2 package (Wickham, 2009), with model predictions extracted using the keepef function from the remef package (version 1.0.6.9; Hohenstein & Kliegl, 2014).

To determine the variance explained by a model, I report two *R* ^2^ statistics for LMMs: marginal and conditional *R* ^2^ (Johnson, 2014; Nakagawa & Schielzeth, 2013). *Marginal R* ^2^ gauges the variance explained by fixed effects, and *conditional R* ^2^ is concerned with the variance explained by both fixed and random effects. Nakagawa and Schielzeth (2013) provided a definition of these measures for LMM and GLMM (generalized LMM) that incorporate random intercepts only. Here, I use Johnson’s (2014) extension for random slopes models, available through the r.squaredGLMM function in the MuMIn R package (version 1.14.0; Bartoń, 2015).

## Results and discussion

The results are presented in five sections. The first three sections focus on presenting the results for the LMMs that included the entire set of image-related as well as the oculomotor and spatiotemporal predictors. These models are referred to as the *full* LMMs; in technical terms, they correspond to the final zcpLMMs derived above. The results for immediacy effects of the local image statistics are presented first, followed by the lag and successor effects (or lack thereof), and finally the oculomotor and spatiotemporal immediacy effects. The fourth section provides *R* ^2^ statistics for the full model, as opposed to the two partial models. The final section is devoted to control analyses.

### Immediacy effects of local image statistics

**Fig. 3:**
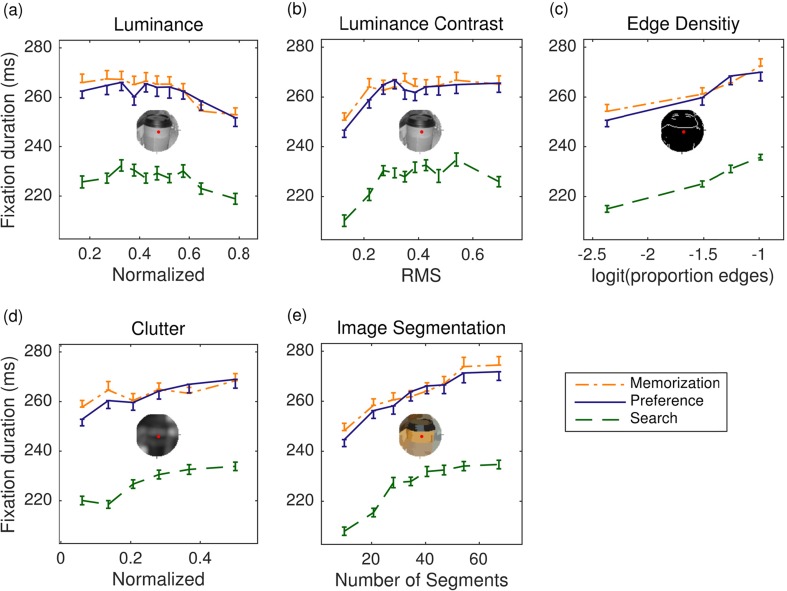
Five main effects of local image statistics on fixation durations for scene memorization (dash-dotted lines), preference judgment (solid lines), and scene search (dashed lines). The dependent variable is the duration of the current fixation *n*. The input variables are (a) luminance, (b) luminance contrast, (c) edge density, (d) clutter, and (e) synergistic segmentation, calculated for 1° circular patches around the current fixation location *n*. Error bars are within-subjects standard errors, using the method described by Cousineau (2005). Data are from the right eye

I first consider the immediate effects of the local image statistics on fixation durations as a function of viewing task. To explore the empirical data, for each image feature and viewing task, the mean fixation duration was calculated as a function of the respective feature. The panels in Fig. 3, one for each feature, display the observed mean fixation durations over suitably binned category means. For each feature, categories were created using quantiles of the continuous variable, resulting in approximately equal-sized data subsets. Figure 3a shows that, for each viewing task, fixation duration decreases with increasing luminance. Thus, brighter fixation locations are associated with shorter fixation durations. The data in Fig. 3b are suggestive of a monotonically increasing relationship between luminance contrast and fixation duration. Furthermore, as the number of edges in foveal vision increases, the fixation duration increases (Fig. 3c). Likewise, as the visual clutter around fixation increases, the fixation duration increases as well (Fig. 3d). Finally, the more meaningful “chunks” are in a patch, the higher the fixation duration (Fig. 3e).

The effects of viewing task were qualitatively similar for each image feature. On the one hand, very similar looking data patterns emerged for the memorization and preference judgment tasks. On the other hand, fixation durations were shorter during visual search than in the two other tasks (Nuthmann et al., 2010), and this does not appear to depend on the respective image feature values.

**Fig. 4:**
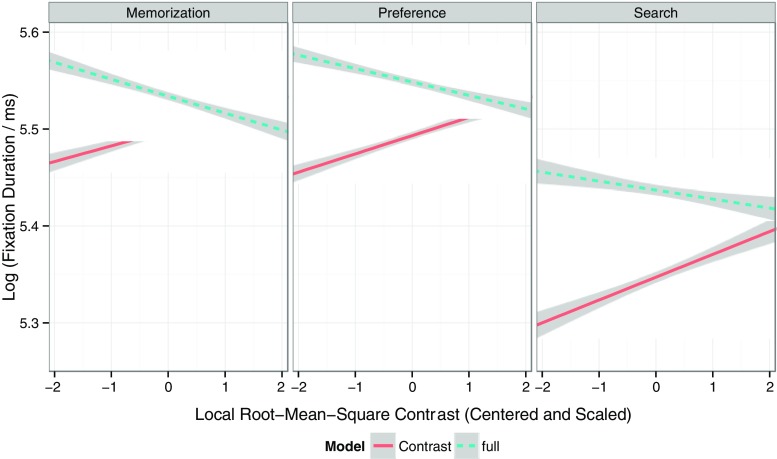
Predicted partial effects of local luminance contrast on fixation durations for the contrast-only model (solid lines) and the full model (dashed lines). Each panel presents data for a given viewing task. Local luminance contrast values are centered and scaled (i.e., *z* scores), and fixation durations are log-transformed. Shaded error bands depict 95 % confidence intervals. See the text for more details

The averaged empirical data in Fig. 3 reflect the main effect of a given image feature by ignoring all other predictors. To illustrate this point, one-predictor LMMs were built. For example, the contrast-only LMM for a given viewing task included contrast as the only fixed effect (in addition to the intercept), as well as uncorrelated by-subject and by-item random intercepts and slopes. In each model, the regression coefficient for the fixed effect of contrast was positive and significantly different from zero (memorization: *b* = 0.016, *SE* = 0.003, *t* = 5.3; preference: *b* = 0.019, *SE* = 0.003, *t* = 6.23; search: *b* = 0.024, *SE* = 0.005, *t* = 4.6). Figure 4 displays the corresponding predicted partial LMM effects, after removing between-subject and between-item variance in the dependent variable. The one-predictor LMMs for the other image features all showed the same pattern of results: The fixed effect of the respective feature was significant, and the sign of the regression coefficient was in agreement with the data depicted in Fig. 3 (i.e., negative for luminance, positive for all other image features).

The question arises whether these relationships would still hold once all predictors were included in the LMM. As was noted earlier, visual features in natural images tend to be correlated for a particular location (Nuthmann & Einhäuser, 2015), and the purpose of LMMs is to factor in the correlations between predictors. The results for the full LMMs are presented in Table 1 (fixed effects) and Table 4 (random effects). For a given viewing task, all immediacy effects of local image statistics on fixation durations were still significant with simultaneous statistical control of all other effects (Table 1). However, the regression coefficient for luminance contrast changed sign. In the contrast-only model it had a positive sign, indicating that higher contrast was associated with *longer* fixation durations. In the full model, however, it had a negative sign, suggesting that higher contrast was associated with *shorter* fixation durations. Figure 4 provides a visualization of this sign switch by depicting the partial LMM predictions from the contrast-only models (solid lines) as opposed to the full models (dashed lines). For the search data, the effect of contrast was only marginally significant in the full model. In summary, the LMM analyses demonstrate that there are *immediacy effects* of local image statistics on fixation durations in scene viewing.

**Table 1:** Linear mixed models fitting log fixation durations for the memorization, preference, and search tasks, fit by restricted maximum likelihood (REML): Means, standard errors, and *t* values of fixed effects on fixation durations

	Scene-Viewing Task								
	Memorization			Preference Judgment			Search		
Fixed Effects	*b*	*SE*	*t*	*b*	*SE*	*t*	*b*	*SE*	*t*
Intercept of mean fixation duration (log)	5.534	0.034	165.02	5.549	0.039	141.8	5.437	0.032	170.37
Local Image Feature Predictors									
Luminance									
Fixation *n*	–0.014	0.004	–3.71	–0.015	0.004	–4.04	–0.023	0.006	–3.57
Fixation *n* – 1	0.004	0.004	**1.09**	–0.003	0.004	**–0.72**	–0.002	0.005	**–0.46**
Fixation *n* + 1	–0.006	0.004	**–1.49**	–0.008	0.004	–2.06	–0.004	0.005	**–0.8**
Luminance contrast									
Fixation *n*	–0.017	0.004	–4.81	–0.014	0.004	–3.9	–0.009	0.005	*–1.89*
Fixation *n* – 1	–0.001	0.003	**–0.32**	–0.004	0.003	**–1.1**	0.002	0.005	**0.42**
Fixation *n* + 1	0.007	0.003	2.09	0.006	0.003	1.97	0.003	0.005	**0.68**
Edge density									
Fixation *n*	0.021	0.004	5.89	0.021	0.003	6.33	0.024	0.006	4
Fixation *n* – 1	0.004	0.003	**1.20**	0.003	0.003	**1**	0.003	0.006	**0.46**
Fixation *n* + 1	–0.005	0.004	**–1.4**	0.003	0.003	**1.05**	–9 × 10^–5^	0.006	**–0.02**
Clutter									
Fixation *n*	0.021	0.005	4.40	0.02	0.005	6.06	0.031	0.007	4.4
Fixation *n* – 1	–0.009	0.005	*–1.88*	–0.001	0.004	**–0.28**	0.001	0.006	**0.09**
Fixation *n* + 1	–0.008	0.005	*–1.74*	–0.002	0.004	**–0.37**	–0.005	0.007	**–0.75**
Number of segments									
Fixation *n*	0.023	0.004	5.79	0.022	0.004	5.77	0.020	0.005	3.77
Fixation *n* – 1	0.004	0.003	**1.01**	–0.002	0.003	**–0.62**	–0.004	0.005	**–0.76**
Fixation *n* + 1	–0.003	0.004	**–0.74**	–0.006	0.003	*–1.76*	–0.004	0.005	**–0.82**
Oculomotor and Spatiotemporal Predictors									
Previous saccade	0.028	0.004	7.06	0.036	0.003	11.55	0.068	0.004	16.09
Previous saccade^2^	–0.011	0.002	–7.01	–0.012	0.001	–8.38	–0.015	0.002	–7.99
Next saccade	–0.025	0.004	–6.85	–0.027	0.003	–7.67	–0.014	0.004	–3.94
ΔAngle	0.065	0.004	18.1	0.067	0.004	17.32	0.021	0.003	6.07
Viewing time	0.04	0.003	13.44	0.045	0.004	12.41	0.058	0.004	13
Viewing time^2^	–0.03	0.003	–10.25	–0.046	0.003	–14.46	–0.046	0.005	–9.21
Central distance	0.004	0.003	**1.64**	0.002	0.003	**0.71**	–0.002	0.004	**–0.38**
*N* of observations	28,442			33,275			14,968		
REML criterion	21,942.6			24,593.9			11,208		

*b* denotes the estimates of the regression coefficients, *SE* the standard errors. **Nonsignificant** coefficients are set in bold (*|t|* ≤ 1.645). *Marginally significant* coefficients are set in italics (1.645 < *|t|* ≤ 1.96)

### Distributed processing: Lag and successor effects

**Fig. 5:**
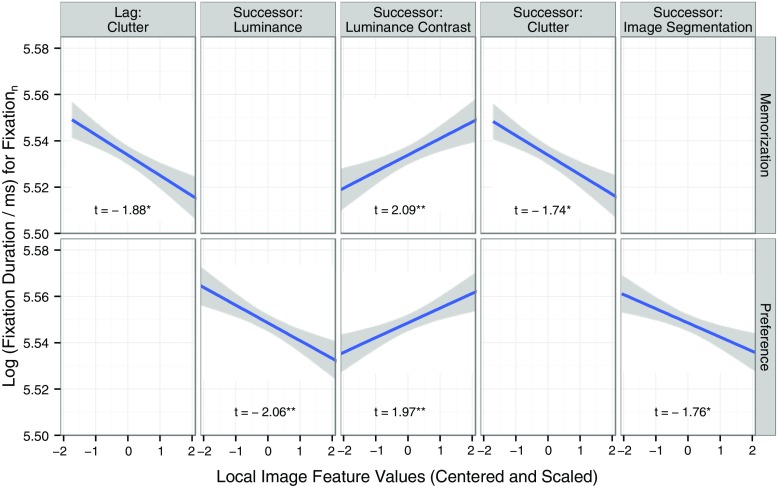
Partial lag and successor image feature effects on log fixation durations during scene viewing. Data from the scene memorization task are depicted in the first row, data from the preference judgment task in the second row. Only significant (**) and marginally significant (*) effects are shown, such that facets with nonsignificant effects are empty

Next, let us consider the image feature predictors that speak to the distributed-processing assumption. Does the duration of the current fixation also reflect the processing demands of the previous fixation location (lag effects due to incomplete processing) and the next fixation (successor effects)?^7^ For the visual search task, there were no lag and successor effects (Table 1), suggesting that processing in this task was not distributed across fixation durations within the visual span. For the memorization and preference tasks, there was some evidence for successor effects and very little evidence for lag effects—see Table 1 and Fig. 5. First, reliable successor effects emerged for the low-level image features. The data from the preference task showed a negative successor effect for local luminance. Thus, the duration of the current fixation *n* was short if the upcoming fixation location *n* + 1 was high in luminance. Moreover, the data from both tasks showed a positive successor effect for local luminance contrast, such that the current fixation duration was long if the contrast around the upcoming fixation location *n* + 1 was high. The regression coefficient for the successor contrast effect had a sign opposite that for the immediacy contrast effect in the full model (Table 1). Second, there were reliable successor effects for the higher-level image features, and there was an interesting task dissociation: The data from the memorization task showed an inverted successor effect for clutter, whereas the data from the preference task showed an inverted successor effect for the number of homogeneous segments. The effects were inverted such that the regression coefficients for the successor effects had signs opposite the regression coefficients for the corresponding immediacy effects. The immediacy effects of clutter and synergistic segmentation were positive, such that high feature values implied difficult processing, and consequently, long fixations. The corresponding successor effects were negative, such that high feature values at fixation *n* + 1 were associated with shorter fixation durations at fixation *n*. Third, there was only one lag effect: During scene memorization, a marginally significant inverted effect of visual clutter at fixation *n* – 1 on the current fixation duration.

**Fig. 6:**
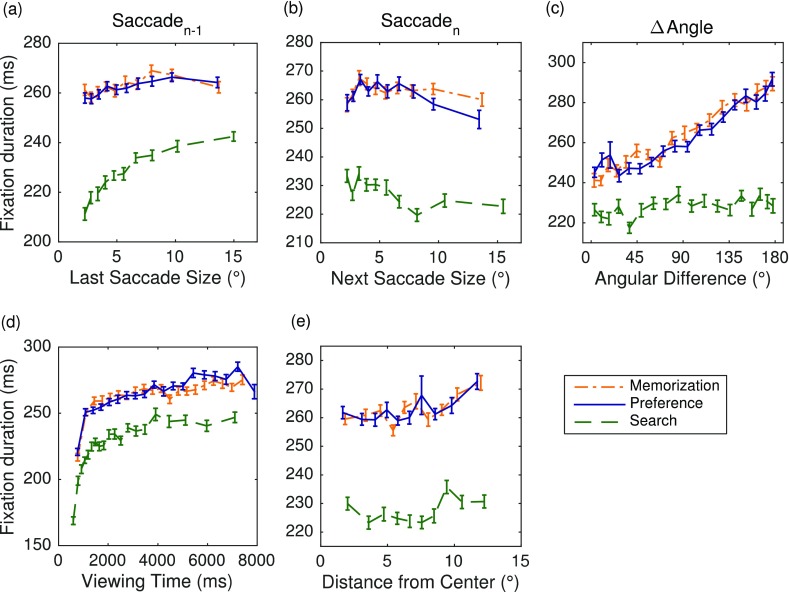
Five main effects of oculomotor and spatiotemporal variables on fixation durations for scene memorization (dash-dotted lines), preference judgment (solid lines), and scene search (dashed lines). The input variables are (a) the amplitude of the last saccade *n* – 1, (b) the amplitude of the next saccade *n*, (c) the angular difference between the two saccades (ΔAngle), (d) the viewing time, and (e) the current fixation’s distance from image center. Error bars are within-subjects standard errors, using the method described by Cousineau (2005)

The LMMs also evaluated whether there were lag effects associated with preview space, represented by the amplitude of the last (or incoming) saccade. For a given viewing task, saccades of larger amplitude were followed by fixations of longer duration. This increase in fixation durations appeared to be less than linear (Fig. 6a). Therefore, the LMMs included both a linear and a quadratic term for the size of the last saccade. Across viewing tasks, the amplitude of the last saccade had a significant positive linear effect and a significant negative quadratic effect on fixation durations (Table 1). Given that the amplitude of the last saccade entered the LMMs as a centered variable, both estimates have clear interpretations independent of each other (Schielzeth, 2010). The positive estimate for the linear term expresses the linear effect of longer saccade amplitudes being associated with longer fixation durations. The negative estimate for the quadratic term substantiates that very long saccade amplitudes elicit lower response values on top of the linear relationship. The amplitude of the last saccade had a particularly strong effect in the search task (Fig. 6a, Table 1).

### Oculomotor and spatiotemporal immediacy effects

The amplitude of the next (or outgoing) saccade showed a significant negative effect on fixation durations, with shorter fixation durations when the next saccade was large (Table 1, Fig. 6b). This effect was stronger in the memorization and preference tasks than in the search task.

The change in saccade direction was measured by coding the angular difference between the last and next saccades (ΔAngle). On the original continuous scale, the values of the input variable ranged between 0° (no change in direction) and 180° (complete reversal). Across viewing tasks, fixation durations increased as the angular difference between the last and next saccades increased (Fig. 6c, Table 1). This positive linear relationship was very strong for the memorization and preference tasks, and weaker (yet significant) for the search task.

For a given viewing task, fixation durations increased as viewing progressed. The effect was particularly strong during early viewing and leveled off during later viewing (Fig. 6d). For a given fixation, the viewing time was calculated as the time (in milliseconds) that had passed between the scene onset and the end of the fixation (Nuthmann & Matthias, 2014). For the statistical modeling, the viewing-time variable was centered and scaled, as were all input variables. The effect of viewing time on fixation durations was modeled by including both a linear and a quadratic term for viewing time in the LMMs (Mills et al., 2011). For each viewing task, there was a significant positive linear effect and a significant negative quadratic effect of viewing time on fixation duration (Table 1).

The final predictor captured the distance of the current fixation from scene center. For a given viewing task, the averaged empirical data in Fig. 6e are suggestive of a slight increase in fixation duration as fixations’ distance from image center increases. However, in the full LMMs, the distance to center had no significant effect on fixation durations (Table 1). In comparison, in the distance-only LMMs the regression coefficient for the fixed effect of central distance was positive and significantly different from zero (memorization: *b* = 0.015, *SE* = 0.003, *t* = 5.13; preference: *b* = 0.017, *SE* = 0.003, *t* = 5.27; search: *b* = 0.009, *SE* = 0.004, *t* = 2.09). The results from the full LMMs demonstrate that this effect was not reliable under simultaneous statistical control of all the other predictors, some of which were correlated with central distance (in particular, ΔAngle).

### Goodness of fit

How much of the variance in fixation durations is explained by the various image feature and nonfeature predictors? For the full models, Table 2 reports two *R* ^2^ statistics for LMMs: marginal and conditional *R* ^2^ (Johnson, 2014; Nakagawa & Schielzeth, 2013). For each viewing task, the variance explained by the fixed effects (marginal *R* ^2^) is above 5 % (5.20 %–6.73 %). This is a good fit, given that the “raw” fixation durations were modeled (Kliegl et al., 2006, for a discussion). The variance explained by both the fixed and random effects (conditional *R* ^2^) is around 20 %. To assess the relative importances of image feature versus nonfeature predictors, the full model for each viewing task was compared to two partial models. The first model was the five-feature distributed-processing model, which included all predictors pertaining to the local image features. The second model was the nonfeature model, which included all oculomotor and spatiotemporal predictors. The results in Table 2 suggest that the marginal *R* ^2^ was larger for the nonfeature model (3.82 %–4.87 %) than for the five-feature distributed-processing model (1.27 %–1.40 %).

**Table 2:** Marginal and conditional *R* ^2^

	Memorization		Preference		Search	
	*R*_LMM(*m*)_^2^	*R*_LMM(*c*)_^2^	*R*_LMM(*m*)_^2^	*R*_LMM(*c*)_^2^	*R*_LMM(*m*)_^2^	*R*_LMM(*c*)_^2^
Five-feature distributed-processing model	1.31 %	12.6 %	1.27 %	15.49 %	1.40 %	13.77 %
Nonfeature model	3.82 %	15.15 %	4.64 %	19.63 %	4.87 %	15.82 %
Full model	5.20 %	17.59 %	6.01 %	22.17 %	6.73 %	19.19 %

To ascertain that the inclusion of local image features in the full model was justified, likelihood ratio tests compared the full model for each viewing task to the corresponding nonfeature model. The full model consistently provided a significantly better goodness of fit than the nonfeature model (memorization: logLik ∆*χ* ^2^(30) = 472.8, *p* < .001; preference: logLik ∆*χ* ^2^(30) = 559.46, *p* < .001; search: logLik ∆*χ* ^2^(30) = 317.05, *p* < .001). The Bayesian information criterion (BIC; decreases with goodness of fit) corrects the log-likelihood statistic for the number of estimated parameters and the number of observations (Schwarz, 1978). For the data from each viewing task, the full model had a smaller BIC than the nonfeature model.

### Control analyses

**Fig. 7:**
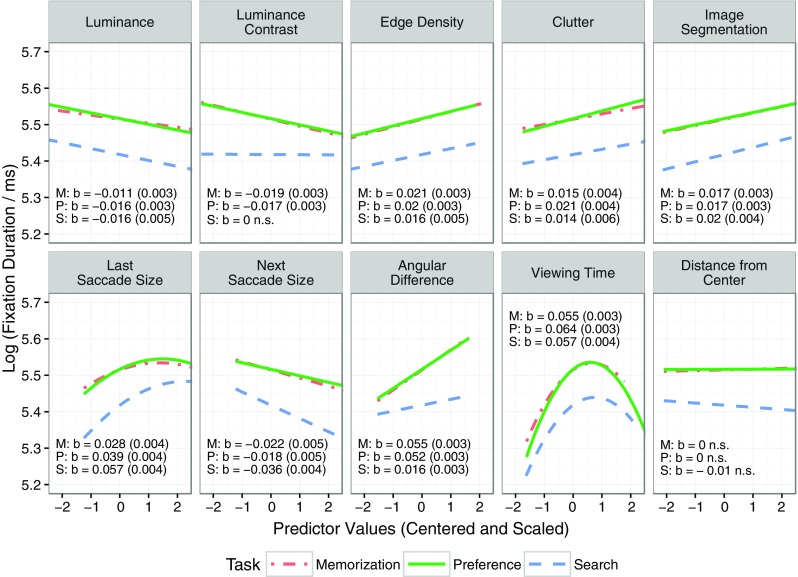
Control analyses of immediacy effects, excluding lag and successor effects. Predicted partial immediacy effects of local image features (top row of panels) and oculomotor and spatiotemporal effects (bottom row of panels) on log fixation durations during scene viewing. Each panel depicts the predictions from the three viewing-task control models. The panels additionally present the coefficient estimates (*b*) and their standard errors (in parentheses) for the fixed effects (linear predictor terms only)

A central goal of the analyses above was to examine the influence of local image statistics on fixation durations at the current, previous, and next fixation locations. Therefore, the analyses reported so far were based on triplets of fixations. The main analyses were complemented by control analyses that excluded the lag and successor effects. Thus, rather than analyzing fixation triplets, the control analyses considered all individual fixations and their incoming and outgoing saccades. The control analyses were run for two reasons. First, they afforded greater statistical power. Second, they allowed for testing whether the immediacy effects of feature and nonfeature variables generalized when fixations with short incoming or outgoing saccades were included. Figure 7 provides a graphical summary of the results. The figure displays the predicted partial immediacy effects of local image features (top row of panels) and the oculomotor and spatiotemporal effects (bottom row of panels) for the three viewing tasks. The panels also present the coefficient estimates (*b*) and their *SE*s (in parentheses) for the fixed effects. The results for the control analyses were very similar to those from the main analyses presented in Table 1. The only exception was the immediacy effect of luminance contrast in the search task: Whereas this effect was marginally significant in the main analysis, it was nonsignificant in the control analysis.

## General discussion

Scene perception involves the interplay of image-related, task-related, and oculomotor processing constraints. An important research question in scene perception is to investigate how these factors influence how long the eyes remain fixated in a particular location. The contribution of the present work is to present an LMM-based statistical modeling framework to simultaneously test the influence of a large set of image-related as well as oculomotor and spatiotemporal variables on fixation durations during real-world scene perception and search. A specific aim was to test how local image-based indexes of processing difficulty influence the fixation durations at the current, previous, and next fixation locations. In addition, the study investigated how the control of fixation durations depends on cognitive top-down influences, operationalized as probing the effects of three different viewing tasks.

### Global and immediate adjustments of fixation durations during scene viewing

Previous researchers have investigated both global and immediate adjustments of fixation durations during scene perception. Examples of global control are the effects of viewing task (Mills et al., 2011; Nuthmann et al., 2010) and the effects that image-wide degradations of low-level features have on fixation durations (Henderson et al., 2013; Ho-Phuoc et al., 2012; Loftus, 1985). Studies in which image-wide feature modifications were employed in a fixation-contingent manner have shown that fixation durations can be immediately adjusted on a fixation-by-fixation basis (Glaholt et al., 2013; Henderson et al., 2013; Henderson et al., 2014; Walshe & Nuthmann, 2014a). The present study offers an important extension, by investigating properties of local control of fixation durations—that is, whether fixation duration varies as a function of the processing difficulty of the currently foveated scene content. Corpus analyses involved a set of five low-level, mid-level, and higher-level visual image features, yielding local image-based indexes of processing difficulty.

### Immediacy effects of local image statistics

The present data are the first to show immediacy effects of local image statistics on fixation durations in various scene-viewing tasks. For the two low-level image features, the LMM immediacy effects were negative, such that low luminance and contrast were associated with longer fixation durations. For the mid-level and higher-level features, the immediacy effects were positive. Specifically, fixation duration increased as the number of edges in foveal vision increased, as the visual clutter around fixation increased, and as more meaningful “chunks” appeared in foveal vision.

Thus, all five image features showed significant immediate effects on fixation durations. The one exception was the effect of luminance in the search task, which was found to be either marginally significant (main analysis) or nonsignificant (control analysis). The present results regarding the “when” decision differ from our previously reported results for the “where” decision (Nuthmann & Einhäuser, 2015). Analyzing data from the memorization task only, GLMMs were used to investigate whether the five image features can independently predict whether or not image patches are fixated. The results suggested that neither luminance nor contrast has an independent effect above and beyond what can be accounted for by edge density and the two higher-level features approximating local object density in the scene (Nuthmann & Einhäuser, 2015). The effects of luminance and contrast on fixation probabilities disappeared when edge density (and clutter) were included in the GLMM. The fixation duration data reported here showed a different picture: When contrast was considered in isolation, the data showed a positive relationship, such that high contrast was associated with longer fixation durations. In this situation, high contrast is a proxy for the presence of edges. As soon as edges are explicitly accounted for in the LMM, the effect of high contrast may be limited to scene regions in which our visual system, on the basis of parafoveal information, expects to encounter edges, but where none exist. This leads to a reduction in processing time, which manifests itself in a negative fixed-effect estimate for luminance contrast. Confirmation of this possibility will require experimental tests with modified stimuli in which contrast and edge density are experimentally decorrelated.

The results for luminance contrast differ from those in previous research, which did not find any systematic relationship between contrast and fixation duration (Einhäuser & König, 2003). Moreover, the immediacy effect of clutter on fixation durations contrasts with a previously reported null effect in a search task (Henderson et al., 2009). The different results may be due to differences in the task requirements. The present search task had observers look for a prespecified object in the scene. In contrast, in the Henderson et al. study, observers searched for small and barely visible letters embedded in photographs of real-world scenes, a task that does not require detailed scene processing.

### Spatially distributed processing

The present analyses also examined whether scene processing is distributed across fixation durations within the visual span. This was not the case for the visual search task (Table 1). For the memorization and preference tasks, some evidence for successor effects was revealed (Fig. 5). A successor effect can be described as orthodox if it has the same direction as the corresponding immediacy effect (cf. Hyönä & Bertram, 2004, for results from reading). Such effects are consistent with the assumption that parafoveal processing difficulty slows down foveal processing. There was one orthodox effect in the present data—a negative successor effect for local luminance in the preference task. The duration of the current fixation *n* was long if the upcoming fixation location *n* + 1 was harder to process, due to a reduction in luminance (Fig. 5). The remaining successor effects can be described as paradoxically inverted, in that the regression coefficients for the successor effects had signs opposite those of the corresponding immediacy effects. Notably, upcoming locations that were particularly informative—characterized by high clutter in scene memorization and a large number of segments during preference judgments—attracted early saccades to themselves, resulting in shorter processing time for the current location. One way to think about this effect is that the properties of the parafoveal upcoming location may serve as “magnets” to draw the eyes to them. This interpretation is in agreement with the idea of parafoveal “magnetic attraction,” which was introduced by Hyönä and Bertram (2004) to account for paradoxical inverted parafoveal-on-foveal (PoF) effects in reading. The positive successor effects for local luminance contrast in the memorization and preference tasks are explained less well by the magnet view, since it is unclear why the eyes should be drawn to low-contrast regions in extrafoveal vision.

There was no evidence of lag effects due to incomplete processing. The logic underlying these effects is that processing difficulty at the previous location may spill over, inflating the fixation duration of the current fixation. For example, high foveal clutter at fixation *n* – 1 should be associated with longer durations of fixation *n*. However, the opposite effect was observed, such that high foveal clutter at fixation *n* – 1 was associated with shorter durations of fixation *n*. This marginally significant inverted lag effect was only observed during scene memorization.

However, across tasks a reliable lag effect was associated with preview space, represented by the length of the incoming saccade. Fixation durations were systematically prolonged as saccade length increased (Table 1, Fig. 6a). If the distance traversed by the eyes during the saccade is long, the previous fixation *n* – 1 yields less preview of the scene region sampled with the current fixation *n*, and this reduced parafoveal processing inflates the duration of fixation *n*. This saccade distance effect provides evidence that parafoveal processing takes place in scene viewing and that it can affect fixation durations. Such a relationship between saccade amplitude and the subsequent fixation duration was not observed in free viewing (Tatler & Vincent, 2008), but it has been found in many reading studies (Angele et al., 2015; Kliegl et al., 2006; Schad et al., 2010; Vitu et al., 2001; Wotschack & Kliegl, 2013).

### Oculomotor and spatiotemporal immediacy effects

The amplitude of the outgoing saccade showed the inverse effect, such that fixation durations were shorter when the next saccade was large (Table 1, Fig. 6b). This contrasts with the null effect described by Tatler and Vincent (2008). It is conceivable that this negative effect may be related to the two modes of visual scene processing that appear to exist (Unema et al., 2005). *Ambient* (or *global*) processing is characterized by shorter fixation durations that are mostly followed by saccades of larger amplitude. *Focal* (or *local*) processing is characterized by longer fixation durations that are mostly followed by small-amplitude saccades. Analyses based on the two viewing modes evaluate saccade amplitude as a function of fixation duration, whereas the present analyses considered fixation duration as a function of saccade amplitude; both analyses describe the relationship between the same two variables, without implying causation.

The data confirm that a change in direction from one saccade to the next comes at a cost. For a given viewing task, the data showed a linear increase in fixation duration as a function of the angular difference between the last and next saccade. Such a relationship has been previously reported for various scene-viewing tasks (for scene memorization, Smith & Henderson, 2009; for scene search, Smith & Henderson, 2011; for free viewing, Tatler & Vincent, 2008; Wilming et al., 2013). In the present study, in which task was manipulated in a within-subjects design, the effect was very strong in the memorization and preference tasks, and weaker in the search task. As was noted in the introduction, the increase in fixation durations for return saccades (ΔAngle = 180°) may have been due to O-IOR, saccadic momentum, or a combination of the two (Klein & Hilchey, 2011, for a review). Dissociating the influences of O-IOR and saccadic momentum requires different analyses, which were the focus of an article by Luke et al. (2014). In brief, Luke et al. compared two subsets of data—pairs of saccades in which the next saccade landed either *within* or *outside* the zone of O-IOR. Above and beyond saccadic momentum, they found additional fixation duration costs for making return saccades only when the next saccade landed within the zone of O-IOR, suggesting that temporal O-IOR and saccadic momentum are independent processes. With regard to task effects, Luke et al.’s analyses suggested that saccadic momentum is task-sensitive, and thus under cognitive control, whereas O-IOR is not.

Given that the spatial decision of where to fixate next is associated with a strong central bias (Mannan et al., 1996; Nuthmann & Einhäuser, 2015; Tatler, 2007; Tatler et al., 2005), the present analyses explored whether the temporal decision about when to move the eyes was influenced by the fixation’s distance from image center. This was not the case, since central distance had no independent effect on fixation durations. Finally, the present data replicate the well-known finding that fixation durations increase over the time course of scene inspection and/or scene search, and that this increase is not purely linear (Antes, 1974; Mills et al., 2011; Pannasch et al., 2008; Unema et al., 2005).

### Effects of viewing task

To directly assess the effect of viewing task for a given (smaller) set of predictors, one would need to specify an LMM that additionally included “viewing task” as a categorical predictor, as well as the interactions between viewing task and the predictors of interest. The approach taken here was to build separate LMMs for the three different viewing tasks. However, the predictors were placed on a common scale by standardizing their units to units of standard deviations. In this case, the sizes of the standardized regression coefficients and their *SE*s give some indication about the strengths of effects across viewing-task models.

The effects of viewing task can be summarized as follows. Mean fixation duration was shorter during search than during memorization and preference judgment. For each viewing task, viewing time was a strong predictor of fixation duration. Overall, similar results were obtained for the memorization and preference tasks. In both tasks, changes in saccade direction had a particularly strong effect on fixation durations (Table 1). There were subtle differences with regard to spatially distributed processing, as was discussed above (Fig. 5). The effects of parafoveal processing manifested themselves differently in search and nonsearch tasks. Despite the large visual span during object-in-scene search (Nuthmann, 2013), no successor effects emerged during search. The lack of successor effects means that the characteristics of the upcoming location did not influence how long the eyes stayed at the current location during search. However, parafoveal processing during search affected fixation durations through a very strong lag effect associated with preview space (this effect was weaker in the other two tasks). At the same time, saccadic momentum was weaker in search than in the memorization and preference tasks (see Luke et al., 2014, for a discussion).

### Implications for computational models

In the reading literature, PoF effects and successor effects are of great theoretical importance (Drieghe, 2011; Murray, Fischer, & Tatler, 2013; Schotter et al., 2012). In essence, these effects are compatible with parallel-processing models like SWIFT (Engbert et al., 2005; Schad & Engbert, 2012), whereas higher-level (in particular, lexical) PoF effects are incompatible with the serial-attention-shift architecture implemented in the E-Z Reader model (Reichle, 2011; Reichle, Pollatsek, Fisher, & Rayner, 1998).

So far, the issue of parallel versus serial processing has received little empirical and theoretical attention in scene perception (Nuthmann & Henderson, 2012). To investigate this issue, the triplet analyses reported here tested for the effects of immediate and spatially distributed (or parallel) processing in scene viewing. In its current implementation, the CRISP model provides a theoretical account for the immediate effects of global scene processing difficulty and the global effects of viewing task on fixation durations (Nuthmann et al., 2010). Building on this work, Laubrock et al. (2013) simulated data from gaze-contingent foveal and peripheral spatial-frequency manipulations. Their model simulations dissociated foveal from peripheral influences on fixation durations, without implementing the underlying oculomotor machinery. The present data provide an empirical basis for computational models of scene viewing to account for immediacy and successor effects of local image features on fixation durations. Importantly, the presence of successor effects depended on the viewing task. In light of these results, a modeling approach that combines global versus local control principles seems a promising way forward (Trukenbrod & Engbert, 2014, in the context of a reading-like scanning task).

### Relative importances of predictors

The present results also speak to the relative importances of feature and nonfeature variables. The standardized regression coefficients were larger in size for most of the nonfeature predictors than for the feature predictors (Table 1). Moreover, the marginal *R* ^2^ was smaller for the five-feature distributed-processing model than for the nonfeature model (Table 2). There are two reasons why this result is less surprising than it may seem. First, this general pattern has also been found in similar analyses of sentence reading data. Nonlinguistic predictors like the size of the incoming saccade, the fixation position within a word, and the word position-in-text (a correlate of viewing time) are particularly strong predictors of fixation times in reading (Angele et al., 2015; Kliegl et al., 2006; Kuperman et al., 2010; Schad et al., 2010). Second, the local indexes of processing difficulty used in the present study were all image-based. Clutter and synergistic segmentation were operationalized as *higher-level*, but not *high-level*, features, since their computation does not include any contextual component (cf. Nuthmann & Einhäuser, 2015). One way to operationalize high-level aspects would be through subjective ratings of “informative” (Antes, 1974; Mackworth & Morandi, 1967) or “interesting/behaviorally relevant” (Onat, Acik, Schumann, & König, 2014) scene regions. Comparing such “interestingness” maps to feature maps, Onat et al. found that interesting locations were associated with longer fixation durations; moreover, interestingness had a larger effect on fixation durations than did the best single low-level feature.

### Outlook

Through the analysis framework presented in this article, I considered the main effects of 20 input variables on fixation durations in real-world scene perception and search. Future research may involve interactions, to test whether more specific hypotheses derived from the distributed-processing assumption generalize from reading (Kliegl et al., 2006; Schad et al., 2010; Wotschack & Kliegl, 2013) to scene viewing.

The approach taken here was to analyze local image features within 1° patches that were centered on each fixation point. The analyses included features that have been proposed as proxies or surrogates of objects in the literature (Christoudias et al., 2002; Rosenholtz et al., 2007). Provided that objects have been annotated, future research may involve object-based LMMs in which the analyses are restricted to fixations that fall on objects in the scenes. One such application would be to investigate immediacy effects of object frequency and predictability (Wang, Hwang, & Pomplun, 2010, for how to obtain these measures) on object-based measures of fixation times, along with the effects of object size and the oculomotor and spatiotemporal predictors considered here. A limiting factor for extending this object-based approach to lag and successor effects is that an exhaustive object-based parcellation of scene images is usually neither available, nor even feasible; an “object” is a hierarchical construct (Feldman, 2003), and objects in real-world scenes oftentimes overlap and occlude each other.

## Conclusion

Recent studies on the “when” decision during scene perception have used image-wide manipulations of individual features to demonstrate that fixation durations adjust to global scene-processing difficulty. The main contribution of the present work has been to *simultaneously* test the *local* effects of various image features on fixation durations at the current, previous, and next fixation locations. For three different viewing tasks, local image features around the current fixation predicted this fixation’s duration. In addition, the amplitudes of incoming and outgoing saccades, the angular difference between the two saccades, and viewing time all had independent effects on fixation durations. The present LMM-based approach provides a powerful tool for understanding scene exploration, because it captures the interplay of image-related, oculomotor, and spatiotemporal variables in controlling fixation durations.

## Appendix

**Table 3:** Dual Latin-square design used in the study

Subject Group	Order	Scene List
1	1 = Search–Mem–Pref	Search: 1, Mem: 2, Pref: 3
2	1 = Search–Mem–Pref	Search: 3, Mem: 1, Pref: 2
3	1 = Search–Mem–Pref	Search: 2, Mem: 3, Pref: 1
4	2 = Pref–Search–Mem	Search: 2, Mem: 3, Pref: 1
5	2 = Pref–Search–Mem	Search: 1, Mem: 2, Pref: 3
6	2 = Pref–Search–Mem	Search: 3, Mem: 1, Pref: 2
7	3 = Mem–Pref–Search	Search: 3, Mem: 1, Pref: 2
8	3 = Mem–Pref–Search	Search: 2, Mem: 3, Pref: 1
9	3 = Mem–Pref–Search	Search: 1, Mem: 2, Pref: 3

Participants were allocated to nine groups, controlling in which order they performed the three viewing tasks and which set of images they were presented in each task. Mem = memorization, Pref = preference

**Table 4:** Linear mixed models fitting log fixation duration for the memorization, preference, and search tasks, fit by restricted maximum likelihood: Random effects and their standard deviations

		Scene Viewing Task		
Group	Random Effect	Memorization	Preference	Search
Scene item	Intercept	2.52 × 10^–2^	3.03 × 10^–2^	3.08 × 10^–2^
Feature, immediacy	*n* luminance	7.86 × 10^–3^	1.29 × 10^–2^	3.54 × 10^–2^
	*n* contrast	1.19 × 10^–2^	1.54 × 10^–2^	0
	*n* edge density	1.20 × 10^–2^	1.00 × 10^–2^	2.52 × 10^–2^
	*n* clutter	1.05 × 10^–2^	1.74 × 10^–2^	2.00 × 10^–2^
	*n* number of segments	1.92 × 10^–2^	1.38 × 10^–2^	1.44 × 10^–2^
Feature, lag	*n* – 1 luminance	1.36 × 10^–2^	1.31 × 10^–2^	1.87 × 10^–3^
	*n* – 1 contrast	5.26 × 10^–6^	1.17 × 10^–2^	1.07 × 10^–2^
	*n* – 1 edge density	0	8.21 × 10^–3^	2.04 × 10^–2^
	*n* – 1 clutter	1.17 × 10^–2^	3.98 × 10^–8^	0
	*n* – 1 number of segments	3.47 × 10^–3^	7.27 × 10^–3^	0
Feature, successor	*n* + 1 luminance	1.41 × 10^–2^	1.73 × 10^–2^	9.93 × 10^–3^
	*n* + 1 contrast	0	9.92 × 10^–3^	0
	*n* + 1 edge density	1.49 × 10^–2^	1.15 × 10^–2^	1.85 × 10^–2^
	*n* + 1 clutter	1.51 × 10^–2^	2.54 × 10^–3^	2.04 × 10^–2^
	*n* + 1 number of segments	1.78 × 10^–2^	1.35 × 10^–2^	6.99 × 10^–3^
Nonfeature	Previous saccade amplitude	2.60 × 10^–2^	1.19 × 10^–2^	1.12 × 10^–2^
	Next saccade amplitude	2.12 × 10^–2^	1.70 × 10^–2^	1.82 × 10^–2^
	ΔAngle	1.34 × 10^–x^	1.13 × 10^–2^	2.99 × 10^–3^
	Viewing time	1.11 × 10^–2^	1.20 × 10^–2^	0
	Viewing time^2^	6.87 × 10^–3^	1.34 × 10^–2^	2.71 × 10^–2^
	Central distance	1.16 × 10^–2^	1.56 × 10^–2^	2.86 × 10^–2^
Scene list	Intercept	5.02 × 10^–2^	6.20 × 10^–2^	5.11 × 10^–2^
Subject	Intercept	9.42 × 10^–2^	1.12 × 10^–1^	8.61 × 10^–2^
	Previous saccade amplitude	1.44 × 10^–2^	1.12 × 10^–2^	9.23 × 10^–3^
	Next saccade amplitude	1.74 × 10^–2^	1.84 × 10^–2^	1.18 × 10^–2^
	ΔAngle	2.15 × 10^–2^	2.58 × 10^–2^	1.33 × 10^–2^
	Viewing time	1.17 × 10^–2^	2.22 × 10^–2^	1.56 × 10^–2^
	Viewing time^2^	3.95 × 10^–3^	1.76 × 10^–2^	0
	Central distance	7.53 × 10^–3^	1.41 × 10^–2^	8.79 × 10^–3^
Subject group	Intercept	3.58 × 10^–2^	2.26 × 10^–2^	1.81 × 10^–3^
Residual		3.48 × 10^–1^	3.42 × 10^–1^	3.40 × 10^–1^

Note that correlations between the random effects were set to 0 to reduce the model complexity

## Footnotes

1 A subset of scenes and targets and the eyetracking setup in this study were identical to the materials and equipment used in the present study.

2 With regard to terminology, a subtle distinction has been suggested in the context of reading research: Distributed-processing effects found in corpus studies using the statistical control approach should be called *lag effects* and *successor effects*, whereas the terms *spillover* and *parafoveal-on-foveal (PoF) effects* should be reserved for evidence obtained with the experimental approach (Angele et al., 2015; Kliegl et al., 2006). The term *PoF effects*, controversially discussed in the literature on reading, implies that they are caused by parafoveal processing. *Successor effect*, in turn, is a less interpretative term.

3 This study does not report inferential statistics.

4 The data from this eye-movement corpus have previously been used to study attentional selection within objects (Nuthmann & Henderson, 2010; Pajak & Nuthmann, 2013) and specific viewing biases during scene perception (Luke et al., 2014; Nuthmann & Matthias, 2014). In contrast, the present analyses assess the influence of local image-based indexes along with oculomotor and spatio-temporal variables on fixation durations, questions that have not been previously addressed.

5 To be precise, whether LMMs test main effects or simple effects depends on the coding scheme used for the categorical predictors.

6 This observation accords well with our previous GLMM analyses of fixation probability, in which the variances for by-item random slopes for image feature predictors were much larger than the corresponding subject variances (Nuthmann & Einhäuser, 2015).

7 By their very nature, successor effects are subtle—that is, smaller than immediacy effects. Therefore, both significant and marginally significant effects are reported.
